# A competence improvement programme for the systematic observation of frail older patients in homecare: qualitative outcome analysis

**DOI:** 10.1186/s12913-022-08328-0

**Published:** 2022-07-22

**Authors:** Torunn Strømme, Ingrid Tjoflåt, Karina Aase

**Affiliations:** SHARE - Centre for Resilience in Healthcare, Faculty of Health Sciences, University of Stavanger, Postboks 8600, N-4036 Stavanger, Norway

**Keywords:** Clinical observation, Competence, Deterioration, Frail old people, Homecare, Improvement project

## Abstract

**Background:**

The growth of frail older patients with extensive care needs in homecare creates a need for competence development. Improvement programmes are essential to fill this knowledge gap. However, the outcomes of such programmes remain unknown. Therefore, the aim of this study is to describe the outcomes of a competence improvement programme for the systematic observation of frail older patients in homecare.

**Methods:**

This study applied a qualitative mixed-method design. Data were collected in two homecare districts using participant observation, focus group interviews, and individual interviews.

**Results:**

The analysis revealed five concepts characterising the outcomes of the competence improvement programme: 1) frequency of vital sign measurements, 2) situational awareness, 3) expectations and coping level, 4) activities for sustained improvement, and 5) organisational issues affecting CIP focus. Substantial differences were revealed across the two homecare districts in how homecare professionals enacted new knowledge and routines resulting from the competence improvement programme. The differences were related to the frequency of vital sign measurements, coping levels, and situational awareness, in which successful outcomes were shaped by implementation issues and contextual setting. This involved whether routines and planned activities were set to follow up the improvement programme, or whether organisational issues such as leadership focus, resources, and workforce stability supported the programme.

**Conclusions:**

This study documents the differences entailed in creating sustainable outcomes of an improvement programme for homecare professionals’ competence in recognising and responding to deteriorating frail older patients. Depending on the implementation process and the homecare context, professionals enact the activities of the improvement programme differently.

**Supplementary Information:**

The online version contains supplementary material available at 10.1186/s12913-022-08328-0.

## Background

The competence requirements for homecare professionals (HCPs) are becoming increasingly challenging due to changes in healthcare. Homecare services are multifaceted, with an increasing number of frail older patients with extensive care needs and complex requirements [1–3]. Frailty is an age-related condition characterised by a decline in physiological capacity and increased vulnerability where patients have a higher risk of rapid deterioration and mortality [4–6]. Failure to recognise and respond to clinical deterioration might result in adverse outcomes, and early recognition by measuring vital signs is emphasised [7]. HCPs often work alone and therefore have a fundamental role in detecting deteriorating frail older patients [8], where observational competence and clinical judgement are vital to providing appropriate patient care [9].

The accelerating growth of the homecare–dependent population creates a need for competence development and new approaches for the care of frail older patients [3, 10]. The competence demands in caring for patients are placed on frontline staff [11]; in homecare they comprise nurses (with bachelor’s degree), skilled health workers (with healthcare education at the upper secondary school level), and assistants (without healthcare education) [12]. Competence refers to an individual professional’s capability and consistency with job demands and the organisational environment [13, 14]. It involves a combination of knowledge, performance, skills, values, and attitudes [15, 16]. Clinical judgement is essential for healthcare professionals; as a problem-solving activity involving assessment and clinical observation [17] and is influenced by factors such as professionals’ education, experience, time constraints, and work unit culture [9].

Homecare is healthcare provided in the patient’s home and entails care for a wide range of patients [18]. Homecare is a comprehensive service including rehabilitative, therapeutic and assistive care, which covers help with tasks such as medications, hygiene, nutrition and clinical procedures. Daily activities are planned according to predetermined work plans, which schedule and estimate the duration of the visits and the tasks required [19].

Competence in homecare has been explored in several studies in terms of the development of standards, competency demands, and self-reports of competence [20–22]. Most studies have focused on the nursing profession, not including the skilled health workers and assistants. In their review, Bing-Jonsson et al. [23] found an imbalance between actual and expected competence in community care. A wide range of competences are expected at an advanced level, ranging from specific tasks in medication management to overarching principles such as safe practice and considerate care. These expected competences may be new to many nurses, especially those with an older degree in nursing; for skilled health workers and assistants, the imbalance between the expected and actual competence might be even higher [23]. In a previous study, we found that the HCPs’ observational competence, including vital sign measurement, varied, and were in many situations insufficient [24].

An improvement programme is essential to fill the knowledge gap in homecare [25], even though implementing new knowledge is difficult [25–28]. Even after successful implementation, adherence decreases over time, and long-term sustainability of improvement initiatives remains challenging [28]. In addressing barriers and facilitators to the implementation of improvement initiatives in primary care, Lau et al. [29] highlighted the importance of and interdependence among the characteristics of the improvement effort, healthcare professionals involved, organisational features, and context. The fit between the improvement effort and the context is seen as essential. Therefore, the aim of this study is to describe the outcomes of a competence improvement programme (CIP) for the systematic observation of frail older patients in homecare.

Two research questions guided the study:

1. How are the outcomes of a CIP in two homecare districts enacted by HCPs?
2. How do implementation and context influence the CIP outcomes?

By outcomes, we refer to the results and impacts of the improvement programme [30, 31]. The outcomes then represent the HCPs clinical judgment, detection, and management of deteriorating frail older patients after the implementation of the CIP. The outcomes also comprise the impact and interrelationship between CIP implementation and the homecare context in which implementation takes place.

### A competence improvement programme

The current study describes the outcomes of a CIP designed and implemented to improve HCPs’ skills in recognising and responding to deteriorating frail older patients. The programme was multi-componential (Table 1) and consisted of a written compendium, a digital learning tool, a teaching day, and simulation-based training. An equipment bag, equipment backpacks, and a form to structure observation, decision-making, and communication were included in the programme [32]. The CIP was implemented during autumn 2017/spring 2018, and data were collected for the current study during autumn 2019/spring 2020.

**Table 1:** A competence improvement programme in homecare

Learning resources	Purpose
Compendium	Theoretical knowledge about systematic observation and communication. The compendium is to be used for learning new subjects and repeating familiar knowledge.
Digital learning tool	Provides opportunities for HCPs to work with the material at any time.
Teaching seminar	Description of the implementation programme. Dissemination of theoretical knowledge in early recognition of deterioration patients in municipal health. Aiming to improve HCPs’ competence.
Skills training	To master vital measurements.
Simulation-based training	Learning objectives: 1) Structured observation using the Airway, Breathing, Circulation, Disability, Exposure (ABCDE) algorithm. 2) Structured communication (ISBAR).
Equipment bag and backpack	To have available equipment for measuring vital signs.
ISBAR form	To structure observation of the patients’ clinical conditions, contribute to decision-making and structure communication in situations when a patient’s clinical condition is changing. The content of the form is the ABCDE algorithm, the ISBAR communication tool, quick Sepsis-related Organ Failure Assessment, Stroke symptoms, National Early Warning Score, and Visual Analogue Pain Scale.

*Note*: See also [32]

## Methodology

A qualitative mixed-method (QUAL-qual) design [33] was used to analyse the outcomes of the CIP in two homecare districts. Participant observation served as the core component of data collection (QUAL); focus group interviews with HCPs and individual interviews with managers and professional development nurses provided the supplemental component (qual).

### Setting

The setting for the study was two homecare districts (A and B) (see Table 2) in two municipalities in western Norway, during their process of implementing and following up the CIP. In Norway as in several other countries, the delivery of homecare is mainly a municipal responsibility, and all inhabitants have the legal right to receive homecare free of charge [19, 34]. The homecare districts were organised as separate departments in each of the municipalities’ healthcare services.

**Table 2:** Overview of the homecare districts

Homecare district	A	B
HCPs:	83	67
• Nurses	31	22
• Skilled health workers	29	30
• Assistants	23	15
Patients	380	300

An ordinary shift in homecare started with the HCPs updating on the patients’ conditions. A report meeting was conducted, where messages and patient statuses were shared, and medications were distributed. Furthermore, the HCPs visited the patients according to predetermined task-oriented work plans. At mid-day, they returned to the office for a break and reports about the patients, new messages, and an update on the remaining tasks of the shift were shared. After the break, the HCPs mainly visited new patients. Some HCPs conducted administrative work in the office. A car was the primary method of transportation between patient visits.

The two homecare districts had different prioritisation of how the different components of the CIP were included in their practices. For example, the organisation of simulation was carried out differently. Nurses and skilled health workers of both homecare districts were included in the CIP. The assistants were additionally involved after a while, as they also visited the patients and needed observational competence to detect deteriorating patients [32].

### Homecare district A

Homecare district A was one of six homecare districts in a city-based municipality. The homecare consisted of two geographical areas, and the HCPs of the homecare were organised into three groups: 1) nurses visiting patients across the geographical areas, 2) skilled health workers and assistants visiting patients in geographical area 1, and 3) skilled health workers and assistants visiting patients in geographical area 2. The latter two groups included a nurse (named a resource nurse) who acted as a supervisor for skilled health workers and assistants and visited patients according to a predetermined workplan. The managers included a department manager with the daily responsibility for the homecare district, and a unit manager who had the overall responsibility for the homecare district and several other health services in the municipality. A professional development nurse worked full-time with competence improvement, improvement projects, and quality improvement for the HCPs.

### Homecare district B

Homecare district B was one of two districts in a large municipality comprising both rural and urban settlements. All HCPs were organised into two groups, including nurses, skilled health workers, and assistants, visiting patients in two geographic areas. The homecare had two department managers having the daily responsibility for each group, and a unit manager having the overall responsibility for the homecare district and another healthcare services in the municipality. Moreover, two professional development nurses worked part-time with competence improvement, improvement projects, and quality improvement for the HCPs. During the study period, homecare district B was reorganised and had a high turnover of managers and nurses. The number of sick leaves was periodically high.

### Recruitment

The CIP was initiated and followed up by a project manager at the Centre for Development of Institutional and Home Care Services (USHT), and the homecare districts were recruited by the centre. The homecare districts and researchers were introduced by USHT. Initially, each homecare and the first author met to talk about CIP implementation and to clarify the roles of the homecare and the researcher.

In both homecare districts, the professional development nurse had overall responsibility for the CIP and functioned as a contact point for the researchers. The criteria for HCPs participating in the study was that they were frontline staff conducting home visits to patients. The sample should also consist of a mix of nurses, skilled health workers and assistants. All managers and professional development nurses were recruited for the study. The managers, in cooperation with the professional development nurse, recruited participants for all parts of the data collection. The scheduled times of the observations, focus group interviews, and individual interviews were sent to the first author after the agreements were settled with the nurses, skilled health workers, and assistants.

### Sample

This study included HCPs (nurses, skilled health workers, and assistants), managers, and professional development nurses. Moreover, the 21 HCPs (11 HCPs in homecare district A and 10 HCPs in homecare district B) were followed during participant observation at their shift (Table 3). HCPs also participated in focus group interviews: homecare district A had 10 HCPs across three focus groups according to their profession, and homecare district B had five HCPs across two focus groups [35]. The focus group size varied between two and five HCPs (see Table 3). All managers and professional development nurses in the two districts were interviewed in semi-structured individual interviews (three in homecare district A and five in homecare district B).

**Table 3:** Sample and data collection in two homecare districts

Homecare district A			Homecare district B		
Sample	Data Collection		Sample	Data Collection	
Nurses (5)	Participant observation (core component)	11 different shifts	Nurses (3)	Participant observation (core component)	10 different shifts
Skilled health workers (4)			Skilled health workers (5)		
Assistants (2)			Assistants (2)		
Nurses (3)	Focus group interviews (supplemental component)	3 focus group interviews	Nurses (3)	Focus group interviews (supplemental component	2 focus group interviews
Skilled health workers (4)			Skilled health workers (2)		
Assistants (3)					
Managers (2)	Semi-structured individual interviews (supplemental component)	3 individual interviews	Managers (3)	Semi-structured individual interviews (supplemental component)	6 individual interviews
Professional development nurses (1)			Professional development nurses (2)		
			Assistant (1)		

### Data collection

The data collection consisted of participant observation (core component), focus group interviews, and individual interviews (supplemental components).

### Participant observation

Participant observation was conducted by following nurses, skilled health workers, and assistants during day or evening shifts in both homecare districts (Table 3) for 4 months (October 2019–January 2020). All observations were completed by the first author. The researcher met during the agreed-upon shift and always asked for the HCP’s permission to accompany them on the shift and highlighted that the HCP could withdraw from the study at any time. All HCPs signed written informed consent forms. The first author engaged in all aspects of a shift, including home visits to patients, car travel between home visits, shift reports, and meetings. This reflected participating in the HCPs’ practices, routines, and work environment, including participants’ communication and reflections [36]. Moderate participation was used in shift reports, meetings, and when the HCPs visited patients in their homes, which means that the researcher was present in the setting but not actively participating [36]. Active participation was used during car transport between the patients’ houses and during lunch breaks; at these times, the researcher engaged in conversations with the HCPs [36].

An observational guide (Supplementary file 1) was used, which focused on systematic clinical observations and how this was performed in the patient’s home, and in discussions and reflections during meetings at the homecare office. Furthermore, the organisational structure of the homecare districts and the collaboration between the HCPs in the homecare was a focus. Simple notes were made during the observations. These notes were written as detailed field notes, which included eyewitness observations, informal and natural conversations, or interviewing descriptions [31], resulting in 138 pages. A total of 75 h of participation across day and evening shifts for homecare district A and 70 h for homecare district B were conducted.

### Focus group interviews

Five focus group interviews were conducted at the homecare office, each with HCPs with similar professional backgrounds (nurses, skilled health workers, and assistants) (Table 3). According to the literature, the focus group size is recommended with five to ten participants [31, 35]. The first author led the conversation in the focus groups, whereas the second or third author observed the interaction, took field notes, and summarised the topics discussed. A semi-structured interview guide was applied with a focus on experiences of the implementation and the outcomes of the CIP guide (Supplementary file 2). The interviews lasted for about an hour. They were tape-recorded and comprised 173 pages of transcripts.

Because of the COVID-19 situation, all the individual interviews and focus group interviews were postponed to May and June 2020 until the situation was clarified and COVID guidelines allowed researchers to visit the homecare districts. This also led to a reduction of participants in the focus group interviews, with two to five HCPs in each group (Table 3). Furthermore, in homecare district B, an individual interview was conducted with one assistant instead of a focus group interview because of the difficulties in recruitment of this professional group.

### Individual interviews

Individual interviews [30, 37] were conducted with managers and professional development nurses (Table 3) at each of the homecare offices. The first author led all the interviews, and a semi-structured interview guide with a focus on the managers/professional development nurses’ experiences of CIP implementation and the perceived outcomes guided the conversation guide (Supplementary file 3). The interviews were tape-recorded and comprised 100 pages of transcripts.

### Analysis

Qualitative content analysis [38, 39] was used to structure the participant observations, focus group interviews, and individual interviews in both homecare districts [33, 40].

The analysis was conducted in the following steps [38, 39]: 1) the transcripts or the raw data were read through several times to become familiar with the collected data, 2) the raw data were open coded with few words or codes covering the content and with a clear connection between each code and the raw data, 3) the codes with common content were grouped into sub-concepts, and the raw data was reviewed to check the data included in the identified open codes, and 4) the sub-concepts were further grouped into five concepts (Supplementary file 4).

The first author led the analysis process, and the three authors held several meetings through all steps to discuss and achieve a common understanding.

The observational data (core component), focus group interviews, and individual interviews (supplementary components) were analysed separately, and then the datasets were combined to produce a description of the findings (Table 4) [33]. The three datasets were written as one descriptive text. The research questions guided the process. The supplemental components added information to the core component and helped the researchers address the research questions from different perspectives [41].

**Table 4:** The qualitative mixed-method analysis process

Aim: To describe the outcomes of a CIP for the systematic observation of frail older patients in homecare settings	
QUAL method	+qual method,
Participant observations	Focus group interviews Individual interview
Qualitative content analysis	Qualitative content analysis
Results narrative of the QUAL+qual	

### Ethics

This study was approved by the Norwegian Centre for Research Data (NSD; no. 54855). All participants were informed of their protected confidentiality and their right to withdraw at any time. All participants provided written informed consent, and the management of both municipalities approved the study. Transcripts were made anonymously by deleting any identifying information, and the participants were guaranteed that the data tapes and transcripts were stored in line with ethical guidelines and would be deleted after study completion.

The first author, who conducted participant observations in the patients’ homes, signed a declaration of confidentiality in both districts. The first author is a healthcare professional (intensive care nurse) directed by both healthcare legislation and expectations towards researcher neutrality [42]. During observation, the researcher may observe situations where a patient was not cared for according to professional regulations. Such situations were discussed with both the other authors and the managers of the homecare districts. In these situations, professional ethics should take priority over researcher neutrality [42], and healthcare professionals would be notified in case of adverse events. In a few situations, the author asked the HCP to be aware of the patients’ clinical situation by measuring the vital signs. No adverse situations arose.

## Results

The analysis revealed five concepts related to the outcomes of the CIP: 1) frequency of vital sign measurements, 2) situational awareness, 3) expectations and coping level, 4) activities for sustained improvement, and 5) organisational issues affecting CIP focus. The results from the two homecare districts were combined and compared in the descriptions of each of the five concepts.

### Frequency of vital sign measurements

The CIP was designed and implemented to improve HCPs’ observational competence, and vital sign measurements was key for early recognition of deterioration of the patient condition. The frequency of vital sign measurements by HCPs was different in the two homecare districts after CIP implementation. The frequency increased in homecare district A, the HCPs experienced an increased focus on measuring vital signs whereas in homecare district B, most HCPs rarely measured vital signs after CIP implementation.

In homecare district A, the increased frequency of vital sign measurements was experienced by the managers as an important outcome of the CIP:

Currently, vital signs are measured at an earlier stage, and clinical observations seem more precise. The situation has changed from HCPs stating that ‘the patient doesn’t feel well’ to more detailed explanations of the patient’s status and situation […]. More HCPs know how to conduct observations. (Individual interview 1, homecare district A, manager)

The patient’s clinical situation was assessed by consistently measuring vital signs for all new patients and in cases of patient falls. The vital signs of all new patients were highlighted as a measure to gain knowledge of their ‘normal’ condition. A nurse described this during an observation:

The nurse experienced that a substantial change had taken place in the homecare district. A routine is in place expecting measurements of normal vital signs for all new patients. She sees this as a very good thing in that HCPs are familiar with what is expected by them and that it makes is easier to collaborate. […] They all seem to think differently and assess the patient’s clinical situation at an earlier stage. The ‘wait and see’ attitude is less visible. (Observation 4, homecare district A, nurse)

Prior to the CIP, in the case of a patient fall, HCPs helped the patient up and checked for pain and injury. After the CIP, vital signs were consistently measured in these situations in homecare district A. They found it important to identify an underlying cause for the fall, which could reflect early deterioration. During a day shift, the following situation occurred:

During the report at the nursing station, a safety alarm for one of the patients is activated. A skilled health worker (who has the patient on her list) drives the car directly to the patient’s house. When arriving at the patient’s apartment, the patient is lying on the floor in her bedroom, probably because of a fall. ‘Here you are, how are you?’ the health worker says, ‘Are you in pain?’ The patient denies having pain, and the health worker states that they will help her. A nurse then arrives at the apartment, wondering how the fall happened. The patient said she thought she slipped on the floor when she got out of bed and did not really fall. She insists that she still wants to go to the day centre. The nurse and the skilled health worker check the patient for injuries and help the patient in a chair. The nurse proceeds to other work tasks, and the skilled health worker helps the patient to the bathroom and then measures the vital signs. […] Respiration rate is 27/min, pulse is 88/min, and blood pressure is 140/83. The skilled health worker reflects on the fact that the respiration rate is high and wonders what to do. The patient is still persistent in his desire to attend the day centre. The skilled health worker then concludes that it should be okay, although it is important to report the change in vital signs and conduct new measurements during the evening shift. (Observation 2, homecare district A skilled health worker)

The patient was admitted to the hospital the same evening after new vital signs were measured by HCPs at the next shift; the respiration rate was still high. The patient was diagnosed as having pneumonia and was treated for a few days at the hospital before being discharged.

HCPs experienced that they were currently more “hands-on” changed patient conditions. The normal vital signs acted as a comparison between the patient’s present condition and normal situation. They thus helped them indicated patients’ deterioration. In homecare district A, the HCPs did not have the normal vital signs available during home visits as they did not have a digital version of the patient’s journal. Normal vital signs were documented in the journal, which was available at the office. The HCPs resolved this by calling the office for information about the patient’s normal situation.

In homecare district B, several HCPs noted that the CIP was inactive, and vital signs were rarely measured when the patients’ condition had changed. During an observation, an HCP described the situation as follows:

The skilled health worker experiences that measuring vital signs is not often required. However, the CIP has been an important input. The skilled health worker smiles a little and expresses that the vital signs should probably be measured more often. (Observation 6, homecare district B, skilled health worker).

There were several situations in homecare district B when changes in patient condition were noted, including confusion, or not feeling well, and vital signs should have been measured to identify possible deterioration and the need to respond to the change. The following situation during an evening shift shows a patient describing a change in patient condition both regarding not feeling well and inhalation without normal effect. All signs of possible deterioration in which vital signs should have been measured:

During a home visit, the nurse is asking the patient, ‘How are you?’. The patient replies that he is not feeling well and has a hard time breathing. He says that the inhaler is not working properly […]. The nurse responds that perhaps the patient should contact the general practitioner […] and ends the conversation by repeating that he should not hesitate to call the HCPs if the deterioration continues. (Observation 7, homecare district B, nurse).

HCPs in homecare district B expressed an uncertainty about when the measurements of vital signs were required. During this evening shift, a patient was very tired, and the skilled health worker experienced a change in patient condition.

During an evening shift, a skilled health worker is visiting a male patient, and we arrive in a dark apartment. […] The patient is lying in bed, and the skilled health worker wonders why he is so tired. It’s only 5 p.m. The patient does not want to get up and replies that he is tired and wants to stay in bed. The skilled health worker tries to persuade the patient to get up to at least get some food. According to the patient, it is not necessary as he has had dinner. The skilled health worker wonders if he is usually that tired. Afterwards, when returning to the car, the skilled health worker reflects whether something is wrong with the patient as he is so tired and perhaps, she should have measured the vital signs. (Observation 1, homecare district B, skilled health worker)

In both situations, the HCPs perceived a change in patient condition but did not measure their vital signs to detect deterioration. Lack of consistency in vital sign measurements were confirmed by managers and professional development nurses. The HCP was responsible for detecting early deterioration during the patient visit. Thus, this was not considered satisfactory, and the CIP needed to be revisited.

### Situational awareness

In homecare district A, other than situation of falls and new patients, no common descriptions of when the HCPs should measure vital signs were laid down. In two situations of a change in the patient’s physical function, one HCP measured vital signs, whereas the other HCP did not. The detection of deteriorating patients thus became random and dependent on the individual HCP. In homecare district B, HCPs described acute patient situations as rare, so they did not think it was necessary to use the ISBAR form and measure vital signs. In homecare district A, a nurse explained that it would be easier in an emergency department at a hospital.

Then, all vital signs were measured regularly. In homecare, we act more based on our ‘instincts’. Measurements are taken only if there are changes in the patient’s condition’. (Observation 11, homecare district A, nurse).

In homecare district A, the HCPs had experience in early detection of deteriorations by measuring vital signs in patients with fall; however, this was not generally applied to other situations of patients’ change in patient condition. The awareness of the patient’s clinical situation was among the HCPs individual, different, and in many situations appeared as delayed. In the following situation, the skilled health worker commented on the patient’s expectorations and coughs and discussed the changed situation with the patient. Systematic observation and vital signs were missing, which could have objectively discovered the patient’s clinical status:

This morning, a skilled health worker visits a patient who needs assistance with morning care and food preparation. The patient is right-side paralysed after a stroke […]. The patient coughs as he gets up from bed. ‘Oh, you are still coughing. Does it seem like the expectoration is loosening up a bit?’ the skilled health worker asks. The patient replies that he is using a soothing cough syrup to help with that. The skilled health worker says that it is important to mobilise the expectoration and wonders whether the patient has been checked by the general practitioner. The patient does not find that necessary. The skilled health worker replies that, at least, they should follow the situation closely. (Observation 2, homecare district A, skilled health worker)

HCPs in both homecare districts consistently involved patients and asked about their subjective view of their clinical condition. However, the HCPs made little use of this information and had an individual and variable response to the patients’ reported clinical situations, and vital sign measurements were missing in several situations.

In the mid-day report, the HCPs reported on the latest visits to the patients and their clinical situation. This was a suitable arena for discussion, while in many reports in both homecare districts, these reflections were missing, and vital sign measurements were suggested only in a few situations. In a report in homecare district A, an HCP described a patient ‘who was delirious and rude – well, there is a change’. The feedback from a colleague was: ‘Well, we need to do our best’. Reflection on the patient’s cognitive change and the question of whether the alteration was an expression of physical change and deterioration, including suggestions for assessment and further actions, was absent. In another report, an HCP described a patient’s changed clinical condition and received responses from colleagues:

During a mid-day report, an HCP described a situation involving a patient with rectal bleeding. The bleeding was declining and seemed stable. Vital signs were measured, and all HCPs discussed the situation and possible signs related to the bleeding. Should the BP be low or high? They expressed uncertainty but concluded that the patient’s general condition should be as normal as possible. The HCP who visited the patient described her as nauseous, dizzy, and with blood in her diapers. The other HCPs highlighted the importance of a low threshold for calling for help, as the situation could rapidly deteriorate and become dramatic. The HCP responsible for the patient should go back and measure a new set of vital signs. […] An assistant then states that she cannot measure vital signs. (Observation 7, homecare district A, report meeting)

### Expectations and coping level

Several HCPs reported that the CIP provided a structure to use in situations when a patient’s clinical condition needed assessment. The ISBAR form, which they carried with them in the equipment bags and backpacks, was available and acted as guidance during clinical observations, as well as in communication with other healthcare professionals. The form clarified expectations of how to measure vital signs, and when used, the HCPs experienced improvements in communication.

In homecare district A, HCPs described increased coping related to situations of changes in patient condition and possible deterioration. Several discussed a feeling of improved self-confidence:

The skilled health worker tells the researcher that she thinks differently, feeling more engaged in clinical situations now. Before the CIP, she was insecure in situations where patients were deteriorating. She used to call colleagues vague in her descriptions of the situation. Prior to the programme, she said that she did not cope well with acute situations. Currently, she knows how to think – and what to do. (Observation 5, homecare district A, skilled health worker)

The skilled health workers in homecare district A especially experienced a higher level of responsibility in measuring vital signs and had increasingly been taking care of deteriorating patients. Prior to the CIP, measuring vital signs was a nurse responsibility, and in situations of changed patient condition, the skilled health workers called a nurse and tried to describe the situation. In general, HCPs work quite autonomously in homecare, and as such, it is important to be able to cope in such situations. The nurses confirmed the view of the skilled health workers and explained that they more frequently took the initiative in measuring vital signs. The managers acknowledged how skilled health workers had increased their responsibilities:

The CIP has demonstrated that skilled health workers possess the proper knowledge and manage to measure vital signs, resulting in increased self-confidence. This is also due to the fact that skilled health workers have detailed knowledge of the patients, as they regularly visit the same patients. (Individual interview 2, homecare district A, manager)

In homecare district B, the HCPs used the ISBAR form infrequently and subsequently measured vital signs differently. Some HCPs could not remember the last time the form was used or when they measured patients’ vital signs. Some explained that they measured vital signs more often when they did not know the patient. However, when the HCP needed to call the general practitioner, the emergency room, or the alarm central, the vital signs were always measured. In the telephone, the vital signs were asked for by the other healthcare professional, and there was an expectation to picture the patient’s situation with the measurements of vital signs. This made them take vital signs before they called.

The nurse talks about a situation where a patient had a swollen foot, and when she visited the patient, she asked the questions she had learned and measured vital signs. She found that the foot was probably colder than the other. She told the patient that she was worried and wanted to call the emergency room. The nurse at the emergency room acknowledged all her assessments. The ambulance then arrived and picked up the patient. The nurse is not sure how the hospitalisation ended or whether it was a deep vein thrombosis. But for her, it was important that the assessment was done, and that the communication worked well. (Observation 4, homecare district B, nurse)

HCPs reported a feeling of increased safety when they used the ISBAR form and that they should have used it and measured the vital signs more often.

The HCPs indicated that the CIP resulted in a common language when the patients had a change in patient condition. Previously, there was often a vague description of the patients’ condition, and after the CIP, there was a concrete description of vital signs in combination with an explanation of the situation. A skilled health worker explained, ‘It is like we are speaking the same language’ (Observation 6, homecare district A, skilled health worker). In both homecare districts, this was especially highlighted in the situations of calling the general practitioner, the emergency room, or the alarm central. Including the vital signs in the description of the patients’ situations made the recipient understand the seriousness of the situation.

The nurse states that she really likes the bags, backpacks, and the ISBAR form. She valued the form as a really good tool. She also finds that the expectations are clear as to when it is necessary to call a doctor. In those situations, the required vital signs are measured, the dialogue with the doctor is clearer, and the patient needs are communicated. (Observation 4, homecare district B, nurse)

In homecare district A, the skilled health workers also experienced that it was now easier to receive help from nurses. They were all clearer and more explicit about the patients’ problems, and it was easier for the nurse to understand the seriousness of the situation and prioritise:

In the car between home visits, the skilled health worker talks about how it is now easier to receive help from the nurses, as well as the frequency of their own calls directly to the doctor. Prior to the CIP, she says that it was sometimes different; one had to provide good arguments for getting help from the nurses. She describes this as “it’s like we are now speaking the same language”. (Observation 6, homecare district A, skilled health worker)

The assistants’ involvement in the CIP and their responsibility for measuring vital signs were previously unclear. Most assistants did not have formal health education, and therefore, their competence in measuring vital signs was low. This situation was discussed during CIP implementation. The two districts chose different approaches for assistants as part of the CIP. In homecare district A, the managers and professional development nurses decided that assistants were not qualified to measure vital signs. In situations with changes in patient condition and possible deterioration, the assistants were expected to notify a nurse. The assistants were included in the simulation to gain knowledge of when to call for help. The assistants described a feeling of safety when the managers were clear about their expectations. In homecare district B, the assistants were allowed to measure vital signs if they thought they managed.

### Activities for sustained improvement

CIP activities were enacted in different ways in the two homecare districts. Homecare district A completed regular planned activities to sustain the focus of the CIP in HCPs’ daily work. These activities included weekly simulations, discussions on measurement of vital signs, and requirements to bring and use equipment bags and backpacks. The activities were highlighted as important by HCPs:

The nurse states that ‘the weekly simulations work as important reminders of measurements and observation of changes. So are the huddle-board meetings, as measurements are often requested there. (Observation 9, homecare district A, nurse).

Weekly simulations were implemented and completed at the homecare office in which groups of HCPs gathered in accordance with their work plans. Simulations were considered an important arena for learning and sharing experiences:

The skilled health worker reflects on the fact that what they now do in the homecare districts is quite different from what they did before the CIP. There is currently an expectation that measurements of vital signs should be taken. The health worker described it as useful, including the simulation sessions. He/she still expresses an understanding of HCPs that are stressed about the simulations. ‘We are not familiar with being observed while working – we work alone most of the time – so it creates a threshold for everyone’s participation’. He/she explains that the simulation focuses on learning and sharing knowledge and experiences, which is really useful. HCPs are slowly becoming more familiar with the simulation setting than their first simulation experience. (Observation 11, homecare district A, skilled health worker).

The repetitions have helped a lot – the importance of doing it over and over again. It is simply not sufficient, with one or two sessions only. To sustain the skills and make HCP secure in measuring vital signs, it takes a few years. (Focus group interview 2, homecare district A, skilled health workers).

Homecare district B integrated simulation in its yearly activity plans, to take place normally in January/February, while simulation sessions had currently not been completed over the last year. Several HCPs missed the simulations and wanted them to be conducted more often. A skilled health worker explained that the simulations were ‘put on hold’, and she thought simulations were crucial to re-establishing the CIP and highlighting the focus on clinical observation. This was confirmed by the professional development nurse, the CIP required to be implemented again, and a focus on simulations should include the newly employed HCP to sustain their competence. Newly employed HCPs are currently informed by coincidence about the CIP. Some explained that they had heard about it but did not know the contents of the programme. This also included HCPs coming back after a leave, newly employed nurses, skilled health workers, assistants, and temporary staff working during vacations and weekends. A nurse experienced a situation in which a newly employed nurse had not received any follow-up:

The nurse states that new employees are not familiar with the CIP. They bring the bag or backpack at their home visits as everyone else, yet they lack knowledge of how to use the equipment. The nurse observed this during a weekend shift, where several HCPs did not know how to use the bag or backpack. Therefore, she concludes that the CIP needs a better and more systematic follow-up. (Observation 10, homecare district B, Nurse).

As a component of the CIP, the homecare districts received bags and backpacks with equipment for measuring vital signs. Several HCPs expressed the importance of having the necessary equipment, which also served as a reminder of the CIP in both homecare districts. The equipment was consistently brought into the car on a shift, but not all HCPs brought it into the patients’ homes. Homecare district A organised a checklist for maintenance of the contents of the bags and backpacks and incorporated this into the scheduled workplans. Homecare district B lacked a system for maintenance of the bags and backpacks. The responsibility for refilling them was unclear and not included in their work plans. Some HCPs made sure that the bags and backpacks were updated, but many did not include this responsibility in their daily work. Thus, the degree to which equipment and forms were in place differed.

In homecare district A, clinical observation was a point of discussion in several meetings. At the patient safety dashboard meeting, all patients were systematically reviewed, and vital signs became a focus area after CIP implementation. The HCPs described the dashboard meetings as an arena for learning and improved knowledge. In homecare district B, this was missing. At the start of the CIP, the programme was discussed at meetings, and simulations were conducted as important reminders. The frequency of these activities decreased, and the priority and focus on the programme were low. Several HCPs expressed that they were not aware of the CIP. At the start of an evening shift, a skilled health worker expressed this lack of focus:

At the evening shift, a skilled health worker tells the researcher that she should have been more updated on the project. The health worker says that there has been a lack of focus on the project over the last year since they had the first simulations. Even though the simulations were both instructive and useful, the homecare district should have focused more on the project throughout the year. (Observation 1, homecare district B, skilled health worker)

### Organisational issues affecting CIP focus

The organisational situation was different in the two homecare districts, which affected their ability to focus on the CIP. Homecare district A focused on organisational needs to integrate simulations into the HCPs’ predetermined workplans, resulting in an alteration of planned shifts to facilitate conducting the simulations. Moreover, expectations of measuring vital signs of new patients and when patients had fallen. In homecare district B, the organisational situation was challenging with a high number of sick leaves, HCP turnover, busy work plans, and reorganisation at the manager level, with several new managers being employed during the study period. The focus on the CIP was low. One manager explained this as follows:

The nursing manager says that the work in the homecare district is currently extremely busy, with high numbers of sick leaves and need for temporary staff. So, activities beyond patient care – issues that require more in-depth focus – are challenging. To put the daily work aside and prioritise other issues is very difficult. (Observation 10, homecare district B, manager).

HCPs experienced full workplans with little time available. One nurse explained,

There is no time available whatsoever for professional development. She refers to a meeting with the current manager, as such development is his/her responsibility. Yet, the nurse does not experience the manager being hands-on in the situation. (Observation 5, homecare district B, nurse).

The HCP turnover was high, which means both vacancies and many new employees who did not know the CIP. There was no strategy for how to involve new HCPs in the CIP. Some of the new HCPs were informed of the content of the CIP by colleagues, but this happened by chance.

During CIP implementation, homecare district B was reorganised, and a management position was refilled several times. A manager explained that she did not know the content of the CIP. She had heard about the programme, but she was not involved in it. She indicated that the CIP is the professional development nurse’s responsibility.

The involvement of the managers differed in the two homecare districts. In homecare district A, the HCPs described receiving support from the managers. The project was prioritised in their daily work. The professional development nurse was especially highlighted as being engaged and important and a driving force in the project. In homecare district B, several HCPs felt that the managers were insufficiently involved in the CIP. The professional development nurse was primarily responsible for the programme, but it was not prioritised in the previous year. The professional development nurse indicated that it was challenging to fulfil the plan of the programme because of the busy and difficult situation at the homecare. The tasks as a professional development nurse were set aside, and most of the working hours were devoted to direct patient contact. The improvement work in the homecare was not sufficient and the professional development nurse described this as frustrating. There was a desire to work systematically with the CIP, but in daily work, it was not a priority: ‘I have taken responsibility for the entire set of planning and implementation of CIP activities. In addition, there are follow-up activities, as the CIP needs maintenance’ (Individual interview 3, homecare district B, professional development nurse).

The HCPs in homecare district B missed information and activities to sustain their focus on the programme.

The nurse expressed that she sees the project as quite important but that the current situation in the homecare district is frustrating with a lot of disturbances. She hopes that the leaders will become more involved in the project over time. (Observation 5, homecare district B, nurse).

## Discussion

This study determined the outcomes of a CIP for the systematic observation of frail older patients in two homecare districts. The findings document different realities regarding observational competence in the two districts two years after CIP implementation. The differences were shaped by CIP implementation in the homecare districts as well as the contextual setting, including whether routines and planned activities were set to follow up the CIP, or whether organisational issues such as leadership focus, resources, and workforce stability supported the implementation of CIP. This confirms what the literature refers to as the ‘know–do’ gap [25], where the relationship between the contextual setting and the successful implementation of improvement efforts constitutes a challenge [25–27, 29, 43].

A vital component of observational competence in homecare is the measurement of the patient’s vital signs. Since the implementation of the CIP, considerable differences have been observed in the frequency and practice of HCPs taking these measurements across the two homecare districts. In homecare district A, nurses and skilled health workers were clearly expected to measure vital signs of new patients and after a patient’s fall. This increased the frequency of vital sign measurements, which led to earlier detection of changes in patient condition and deterioration. However, besides new patients and cases of falls, the degree to which vital signs were measured when a change in patient condition was noticed was variable. By contrast, in homecare district B, the frequency of vital sign measurement continued to remain low after CIP. Several HCPs considered the need for measurements in homecare as generally redundant. Their knowledge of when to measure vital signs appeared to be low, consistent with the literature pointing to professionals’ reduced autonomy, independence, inability to practise to full scope, and lack of confidence as barriers to the implementation of improvement efforts [29]. Furthermore, the daily activities of HCPs in homecare district B seem to have been driven primarily by predetermined task-oriented work plans, whereas new routines systematising observational competence seem to have had a positive impact on the work practices in homecare district A. These processes of formalisation of knowledge appear to encourage decision-making and remove uncertainty among the HCPs [8].

Beyond the routines related to measurement of vital signs for new patients and cases of fall, there is a need to consider additional routines for changed clinical conditions such as confusion, restlessness, cognitive changes, and physical changes. Clearly defined routines for only very specific clinical conditions support rule-based decisions, which have limitations and may not be applied in situations that go beyond the scope of the routine [44]. This is the case when a patient has a more diffuse change in condition that might evolve over time. Such situations require reasoning and understanding. Cappelletti et al. [9] described clinical decision-making as a movement from understanding to action. Furthermore, decision-making is a cognitive skill in need of different strategies for action, and in both the homecare districts of this study, the movement from knowledge to actual action was influenced by factors such as experience, educational level, working routines of the units, and time pressure [9, 44].

In homecare district A, weekly simulations, routinised measurement of vital signs for new patients and with patient falls, and discussion of patients’ deterioration and changed clinical conditions at huddle-board meetings all ensured sustained knowledge following the CIP. CIP activities were integrated into the existing weekly activities and included in the homecare district’s work plans, and leadership focus was sustained. In this case, knowledge translation took place as a new practice was embedded into routines and no longer challenged [45]. Additionally, the CIP was experienced as important and gave HCPs increased competence in detecting deteriorating patients. The CIP clarified expectations of how to measure vital signs and resulted in a feeling of increased coping levels. In homecare district B, the CIP gradually received low priority, the implementation became inactive, and HCPs were not engaged. This highlights an important difference between the two homecare districts in that the characteristics of the implementation process influenced the outcomes [29, 43]. The CIP required an ‘active process’ in which the individual HCPs were engaged in sustained activities to achieve results (43, s.3). Lau et al. [29] state that the implementation process involves how the improvement initiative is integrated into the exciting workflow of the organisation, how it gains relevant benefits, and how it promotes patient safety.

Contextual factors are also significant mechanisms affecting the changes induced by improvement initiatives [46–49]. The negative outcomes in homecare district B were explained by organisational issues, such as lack of leadership involvement, low workforce stability, and limited resources. The situation changed during the implementation period, and managers explained that focusing on ‘activities beyond patient care – issues that require more in-depth focus’ were challenging. Successful outcomes are as such dependent on an adaption of the improvement measure and a sufficient fit with the context [29, 43]. Stability in leadership positions crucially supports the implementation processes in primary care health services [29, 50], and contextual elements are vital in quality improvement initiatives [50, 51].

### Limitations

The researchers’ presence and role may have influenced the study participants. In particular, the first author was present in both homecare districts at regular intervals for several years and was therefore associated with the CIP by several HCPs. This may have influenced the responses given by the participants, who may have tried to adjust them to what they thought was appropriate. This possible bias has been addressed by using a mixed-method design, including interviews and conversations with HCPs, as well as real-life observations of their work practice. The first author’s specialisation in nursing might also have influenced the HCPs’ practice during observation. This was mitigated by not mentioning this background unless it was asked for. The nursing background further eased entry into homecare and was seen as an essential component in understanding the activities during home visits.

The study was conducted in two homecare districts in two municipalities in Norway, thereby precluding generalisability of our results. Nevertheless, detailed descriptions of the methods and the consequent richness and variety of results might guide readers and future researchers to relate the results to other homecare contexts [30, 51].

## Conclusion

This study documents the differences entailed in creating sustainable outcomes of an improvement programme for HCPs’ competence in recognising and responding to deteriorating frail older patients. Depending on the CIP implementation process and the homecare context, HCPs enact the activities of the improvement programme differently. More specifically, in one of the homecare settings, vital signs were measured more frequently after CIP implementation, activities were established to sustain an increased focus on patient deterioration, and perceptions of an improved coping level among HCPs were common. Nevertheless, after 2 years, differences remained in situational awareness among HCPs and how they understand deterioration. In the other homecare setting, despite an increased expectation of measuring vital signs, they were continued to be measured infrequently. No activities were implemented to sustain the CIP, and organisational issues such as lack of routines, leadership involvement, resources, and workforce stability hindered a focus on competence improvement.

More research, both qualitative and quantitative is required to establish knowledge of the conditions predetermining successful outcomes of observational competence improvement in homecare. Longitudinal qualitative research in different settings and contexts can further our understanding of how HCPs engage in improvement activities and how they are influenced by implementation processes and contextual factors. Observational studies of homecare practices are especially important as they better grasp the “work-as-done” as opposed to the “work-as-explained”. Quantitative surveys with HCPs self-reports of observational competence can furthermore measure the impact of improvement efforts in homecare. Combining surveys with observational studies in mixed methods designs will further expand this relatively new research field.

## Supplementary Information

**Additional file 1.**

**Additional file 2.**

**Additional file 3.**

**Additional file 4.**

## Data Availability

The datasets generated and/or analysed during the present study are not publicly available due to restrictions regarding individual privacy; however, anonymised data are available from the corresponding author upon reasonable request.

## Abbreviations

HCP: homecare professional
CIP: Competence improvement programme
ISBAR form: a form made to structure observation of the patients’ clinical conditions
