# Supplementary material for: A competence improvement programme for the systematic observation of frail older patients in homecare: qualitative outcome analysis
Source: BMC Health Serv Res. 2022 Jul 22;22:938. doi: 10.1186/s12913-022-08328-0 (PMC9303045; doi:10.1186/s12913-022-08328-0)
Supplement: Supplementary file 1 — Additional file 1. [file 12913_2022_8328_MOESM1_ESM.docx]

# Observational guide – Outcome of the CIP

The practices of home care professionals’ (HCP) observational competence

1. Planning of the work shift
   1. Reading written reports
   2. Attending the report meeting
   3. Equipment preparation
2. The HCP’s patient visit
   1. Communication
   2. Systematic observation
      1. Assessment of change in patient condition, e.g., confusion, restlessness, cognitive changes, physical changes
      2. Assessment of the vital signs, e.g., respiration rate, pulse, and blood pressure
      3. Other observations, e.g. blood sugar and urine stix
      4. Use of the ISBAR form, the ABCDE principles, the NEWS score tool, and the ISBAR tool
      5. Use of the available equipment in the bags and backpacks.
   3. Tasks performed
   4. Documentation of the observations
3. Reflections and discussions on deteriorated patients and clinical observations
   1. With other colleagues during the work shift
   2. In meetings or in reports
4. Communication with
   1. Patients
   2. Colleagues
   3. Other health care professionals outside the homecare districts (e.g. calls to general practitioners or the hospital).
5. Organisational issues
