# Supplementary material for: A competence improvement programme for the systematic observation of frail older patients in homecare: qualitative outcome analysis
Source: BMC Health Serv Res. 2022 Jul 22;22:938. doi: 10.1186/s12913-022-08328-0 (PMC9303045; doi:10.1186/s12913-022-08328-0)
Supplement: Supplementary file 2 — Additional file 2. [file 12913_2022_8328_MOESM2_ESM.docx]

# Interview guide: Focus groups with homecare professionals (HCPs: nurses, skilled health workers, and assistants)

# Competence Improvement Programme (CIP) on observational competence

1. Tell us briefly about yourselves (age, formal education, experience)
2. Overall – what do you think about the CIP?
3. Can you tell us about your experiences with the CIP?
   - Which parts of the programme have you participated in?
   - Experiences with the different learning resources of the programme (compendium, teaching seminar, skills training, simulation-based training, equipment bag and backpack, ISBAR form)
   - Implementation of the CIP (what worked/did not work?)
   - Involvement in the CIP process?
4. How do you experience the outcomes of the CIP?
   1. For the practices in the homecare district?
      - Routines related to clinical observation?
      - Changes related to how HCPs work with clinical observation and detection of deteriorating patients? If so – how?
      - What has worked/not worked?
   2. For you as HCPs?
      - The way you reflect on clinical observation and reasoning of the patients? If so, how?
      - How do you consider your observational competence? Any changes after the CIP?
      - Do HCPs have the competences needed to detect deterioration?
   3. For the patients?
      - Has the CIP affected the care/treatment of the patients? If so, how?
      - Experiences/examples related to situations with deteriorating patients?
5. In your experience, has the CIP been integrated into the homecare district’s daily work of the HCPs?
   - Why?
   - What are the barriers and facilitators?
   - What is necessary for the successful integration of the CIP?
   - How can you as HCPs contribute to these changes or not?
   - The managers’ involvement in the CIP?
6. Overall – has the CIP been a success?
   - Why?
7. To summarise your discussion, you have talked about the following issues. Anything you would like to correct or add to the discussion?
