# Supplementary material for: A competence improvement programme for the systematic observation of frail older patients in homecare: qualitative outcome analysis
Source: BMC Health Serv Res. 2022 Jul 22;22:938. doi: 10.1186/s12913-022-08328-0 (PMC9303045; doi:10.1186/s12913-022-08328-0)
Supplement: Supplementary file 4 — Additional file 4. [file 12913_2022_8328_MOESM4_ESM.docx]

| **Supplementary File 4. Example of analysis of observational data to the concept “vital signs are measured more frequently”** | | | |
| --- | --- | --- | --- |
| **RAW-DATA** | **GROUPED CODES** | **SUB-CONCEPTS** | **CONCEPTS** |
| During the report at the nursing station, a safety alarm for one of the patients is activated. A skilled health worker (who has the patient on her list) drives directly to the patient. When arriving at the patient’s apartment, the patient has fallen and is lying on the floor in her bedroom. ‘Here you are, how are you?’ the health worker pronounces. ‘Are you in pain?’ The patient denies having pain and the health worker states that they will help her. A nurse then arrives at the apartment wondering how the fall happened. The patient says she think she slipped on the floor when she got out of bed and did not really fall. She insists that she still wants to go to the day centre. The nurse and the skilled health worker check the patient for injuries and help the patient into a chair. The nurse proceeds to other work tasks and the skilled health worker helps the patient to the bathroom and then measures the vital signs. […] Respiration rate is 27/min, pulse 88/min and blood pressure 140/83. The skilled health worker reflects on the fact that the respiration rate is high and wonders what to do. The patient is still persistent in the desire to attend the day centre. The skilled health worker then concludes that it should be okay, although it is important to report the change in vital signs and conduct new measurements during the evening shift. (Observation 2, Homecare District A skilled health worker) | Patient has fallen. Measuring vital signs | Measuring vital signs in situations when a patient has fallen | Vital signs are measured more frequently |
| The nurse says that we are now going to visit a patient who lives alone in an apartment. He does not take good care of himself and drinks a lot of alcohol. In the car on the way to the patient, the nurse says that last week, she found him on the floor. This patient is on blood-thinner medication, and a fall may be a risk. The nurse tells she took measurements, which were normal – but it seemed like he had hit his head. The nurse called the emergency room, and admission for observation at the hospital was recommended. […] The nurse tells -that in the situation, it was very nice to have the measurement. It is a supportive system that is actual and accurate. Although the situation was quite unclear, the nurse was ensured that home care had an overview. The patient was admitted to the hospital for observation and returned home the next day. (Observation 8, Homecare District A, nurse) | A patient who had fallen and nurse took measurements |  |  |
| It is so we speak the same language to a greater extent. As a skilled health worker, I now measure the vital signs and can thus be more aware of the patient’s situation. For example, I came to a patient who had fallen and took the measurements – as we are supposed to do. Now – they always measure the vital signs when patients have fallen. Why? Yes, it’s probably because we do not know the cause of the fall. […] The patient was admitted to the hospital. […] Previously, it would never have happened. Then we just picked up the patient – and now, the patients receive treatment at a much earlier stage. (Observation 6, Homecare District A, skilled health worker) | Always measure vital signs when patients have fallen |  |  |
| Yes, now we must document the patients’ normal vital signs. In situations such as earlier today, when the patient’s situation has changed, we must compare it with the patient’s normal vital signs. (Observation 5, Homecare District A, skilled health worker) | Measuring normal vital signs | Measuring vital signs for all new patients |  |
| The nurse experienced that a substantial change had taken place in the homecare district. A routine is in place expecting measurements of normal vital signs for all new patients. She sees this as a very good thing in that HCPs are familiar with what is expected by them and that it makes is easier to collaborate. […] They all seem to think differently and assess the patient’s clinical situation at an earlier stage. The ‘wait and see’ attitude is less visible. (Observation 4, Homecare District A, nurse) | Routine on all new patients |  |  |
